# Characterisation of *Salmonella* Typhimurium from a fatal equine nosocomial outbreak and retrospective analysis of equine clinic salmonellosis cases (2010–2025)

**DOI:** 10.1038/s41598-026-40617-0

**Published:** 2026-02-18

**Authors:** Katalin K-Jánosi, Anita Sztojka, István Emil Kis, Imre Biksi, Zoltán Bakos, Eszter Kaszab, Tünde Mag, Ervin Albert

**Affiliations:** 1 Department of Pathology, University of Veterinary Medicine Budapest, Üllő, Hungary; 2 Department and Clinic of Equine Medicine, University of Veterinary Medicine Budapest, Üllő, Hungary; 3 Department of Microbiology and Infectious Diseases, University of Veterinary Medicine Budapest, Budapest, Hungary; 4 National Laboratory of Infectious Animal Diseases, Antimicrobial Resistance, Veterinary Public Health and Food Chain Safety, University of Veterinary Medicine Budapest, Budapest, Hungary; 5 Faculty of Health Sciences, One Health Institute, University of Debrecen, Debrecen, Hungary; 6 Department of Laboratory of Bacteriology, Mycology, and Parasitology, National Centre for Public Health and Pharmacy, Budapest, Hungary; 7 Centre for Metagenomics, Multidisciplinary Health Industry Coordination Institute, University of Debrecen, Debrecen, Hungary

**Keywords:** Equine, Salmonellosis, High case fatality, *Salmonella* Typhimurium, ESBL, WGS, Diseases, Microbiology, Molecular biology

## Abstract

In 2024, a highly fatal outbreak of equine salmonellosis occurred in a Hungarian equine referral hospital, resulting in the death or euthanasia of four out of five affected horses. *Salmonella* (*S.*) *enterica* subsp. *enterica* serovar Typhimurium was identified as the primary causative agent from equine faecal, reflux, and post-mortem intestinal content samples, while one case involved *S.* Coeln. Extensive environmental sampling during the outbreak also yielded multiple *Salmonella* serovars. Whole-genome sequencing revealed a high degree of genetic relatedness among the *S.* Typhimurium isolates, confirming nosocomial transmission. The source of the isolated *S.* Typhimurium was most likely a 3-year-old gelding imported immediately before the admission to the hospital. The isolates belonged to sequence type ST376 and exhibited multidrug resistance, including extended-spectrum β-lactamase and fluoroquinolone resistance genes. Retrospective analysis of microbiological records from 2010 to mid-2024 identified 23 *Salmonella*-positive equine cases involving eight serovars and three probable nosocomial clusters preceding the 2024 outbreak. Following the outbreak, enhanced passive surveillance was implemented between October 2024 and August 2025. During this period, 56 at-risk horses were examined using selective bacteriological testing of clinical and post-mortem samples, of which 11 (19.6%) were *Salmonella*-positive, representing eight different serovars. A distinct cluster of *S.* Martonos was detected, and six of the surveillance-associated cases resulted in fatal outcomes. These findings demonstrate that *Salmonella* is repeatedly introduced into the equine hospital environment and that serovars differ markedly in virulence and transmission dynamics. The exceptionally high case fatality observed during the 2024 outbreak underscores the importance of integrated genomic surveillance, rapid diagnostics, and sustained infection control measures to mitigate the risk of severe nosocomial salmonellosis in equine clinics.

**Supplementary Information:**

The online version contains supplementary material available at 10.1038/s41598-026-40617-0.

## Introduction

Salmonellosis is a bacterial enteric or systemic infection caused by members of the *Salmonella (S.)* genus^1,2^. *S. enterica* subspecies *enterica* is the most important member of the genus and responsible for more than 99% of the *Salmonella*-related diseases of mammalian species^1^. This bacterium can cause infection in all warm-blooded animals including equids^1,3,4^ and also has significant zoonotic importance^5^.

During salmonellosis outbreaks in veterinary hospitals^6–11^ several *Salmonella* serovars have been identified, like *S.* Heidelberg^12–14^, *S.* Infantis^9,14,15^, *S.* Enteritidis, *S.* Newport^6^, *S.* Thompson^15^, *S.* Bareilly, *S.* Kerfeld^16,17^ with *S.* Typhimurium being most frequently detected^6,12,14,15,18,19^, while *S.* Kentucky is also an emerging clone worldwide^20^. In adult horses, *S.* Typhimurium^6,19,21,22^ is the most commonly identified pathogen in nosocomial outbreaks^6,7,19,23,24^ and infections could be associated with asymptomatic carriage to mild febrile disease and severe toxic enterocolitis which may progress to systemic inflammatory response syndrome and subsequent death^1,3,19,25–28^.

The classical triad of clinical signs (fever, leukopenia and diarrhoea)^27^ as well as bacterial shedding in faeces and reflux usually presented in salmonellosis of adult equines. Several risk factors have also been identified, e. g.: abdominal surgery^25,29,30^, which may contribute to disease onset and progression^1^.

The hospital environment itself can serve as a nosocomial source of infection for extended periods, as *Salmonella* serovars are capable of persisting in the surroundings for years, despite the implementation of strict preventive measures^1,7,30^.

In general, the onset of clinical signs after 72 h or more after admission, or the immediate onset of those after a surgery on the first two days of hospitalisation^31,32^, or the recovery of identical serovars from the diseased animals are g nosocomial infections^32,33^. To identify *Salmonella* shedding in horses admitted to a veterinary clinic with colic signs, the presence of fever and the development of reflux during hospitalisation serve as strong predictors^4^.

In veterinary epidemiological practice, large-scale laboratory testing of clinical and environmental samples can be implemented to ensure the absence of cases, however this method strongly depends on the accuracy of sampling and laboratory testing procedures^34^. Faecal culturing (three to five samples, 12–24 h apart) is considered the gold standard for confirming *Salmonella* shedding^28^. Although rapid Polymerase Chain Reaction (PCR) techniques detect only the genetic material of the bacterium^35^, animals positive by PCR should be considered infectious and isolated if possible.

Whole-genome sequencing (WGS) data of the isolates provide the basis for multilocus sequence typing (MLST) and antimicrobial resistance gene (ARG) characterisation, both of which are widely applied techniques in the epidemiological investigation of *Salmonella* spp. outbreaks^6,7,10,13,22,32,36–38^.

The onset and progress of the disease are also determined by virulence factors of the bacterium such as that of the Secretion Systems identified in *S.* (T1SS, T3SS, T4SS and T6SS)^39^, virulence plasmids, fimbriae or flagellae and other elements localised in the chromosome or on *Salmonella* Pathogenicity Islands (SPIs)^1,21,39^. Altogether, 24 SPIs have been described, each contributing to the pathogenicity of the bacterium in the host to a varying degree^39^. The *shdA* gene of *S.* Typhimurium is located on an intergenic region at centisome 54 (CS54)^40^ and it has important role in the colonization of the caecum, the Peyer’s patches and the mesenteric lymph nodes^41^ thus contributing to the persistent shedding of the bacterium in mice^42^. Certain insertion element remnants suggest that mobile genetic elements should be responsible for the distribution of the CS54 island within the genus *Salmonella*^41^.

The increasing onset of antimicrobial resistance poses serious public health concerns^5^, since the most commonly applied antibiotics in horses are critical in human medicine^43^, and the distribution of antimicrobial resistance mechanisms has been identified in *Salmonella* isolates of equine origin^22^. Recently, the horse-to-horse dissemination of extended-spectrum β-lactamase (ESBL)-producing *S. enterica* serovars has been described, highlighting the potential role of the horses as reservoir for this zoonotic agent^32^.

The aim of the authors was to conduct molecular epidemiological analysis of *S.* Typhimurium isolates of a highly fatal outbreak in an equine referral hospital and to identify the primary case of it. Following the outbreak, the authors reviewed the records of microbiological laboratory examinations (January 2010—July 2024) carried out by the Livestock Diagnostic Centre (LDC), in order to assess the *Salmonella-*positive horse cases at the clinic and to identify potential previous *Salmonella-*related case clusters. In addition, enhanced passive surveillance was initiated after the outbreak to investigate the presumed *Salmonella-*carriers and shedders, and to determine the importance of the salmonellosis in equine gastrointestinal diseases observed at the clinic during this period.

## Results

### Data and clinical samples of patients associated with the outbreak

The outbreak was considered to proceed between the admission of the index case (horse #1; 27 July 2024) until the recovery of the only surviving animal (horse #4, 11 September 2024)^44^. During the outbreak all affected horses (horses #1-#5) had a history of acute signs of colic with varying severity at the time of admission to the University Equine Clinic (University of Veterinary Medicine Budapest, Üllő, Hungary). All horses underwent abdominal surgery on the first or second day of their clinical stay and developed clinical signs of salmonellosis within 24–48 h after the abdominal surgery. Four (horse #1, #2, #3 and #5) out of the five horses succumbed or had to be euthanised.

Four days prior to the outbreak, a 3-year-old Dutch Warmblood gelding (horse #0) was admitted to the clinic with clinical signs of suspected transport-associated pneumonia. The animal had been isolated at the time of admission, and intensive care was started. On the night of admission, the animal started showing severe signs of colic and succumbed by the next morning.

Summarized data of patients and clinical records are shown in Supplementary Material.

### Pathology and histopathology

**Fig. 1:**
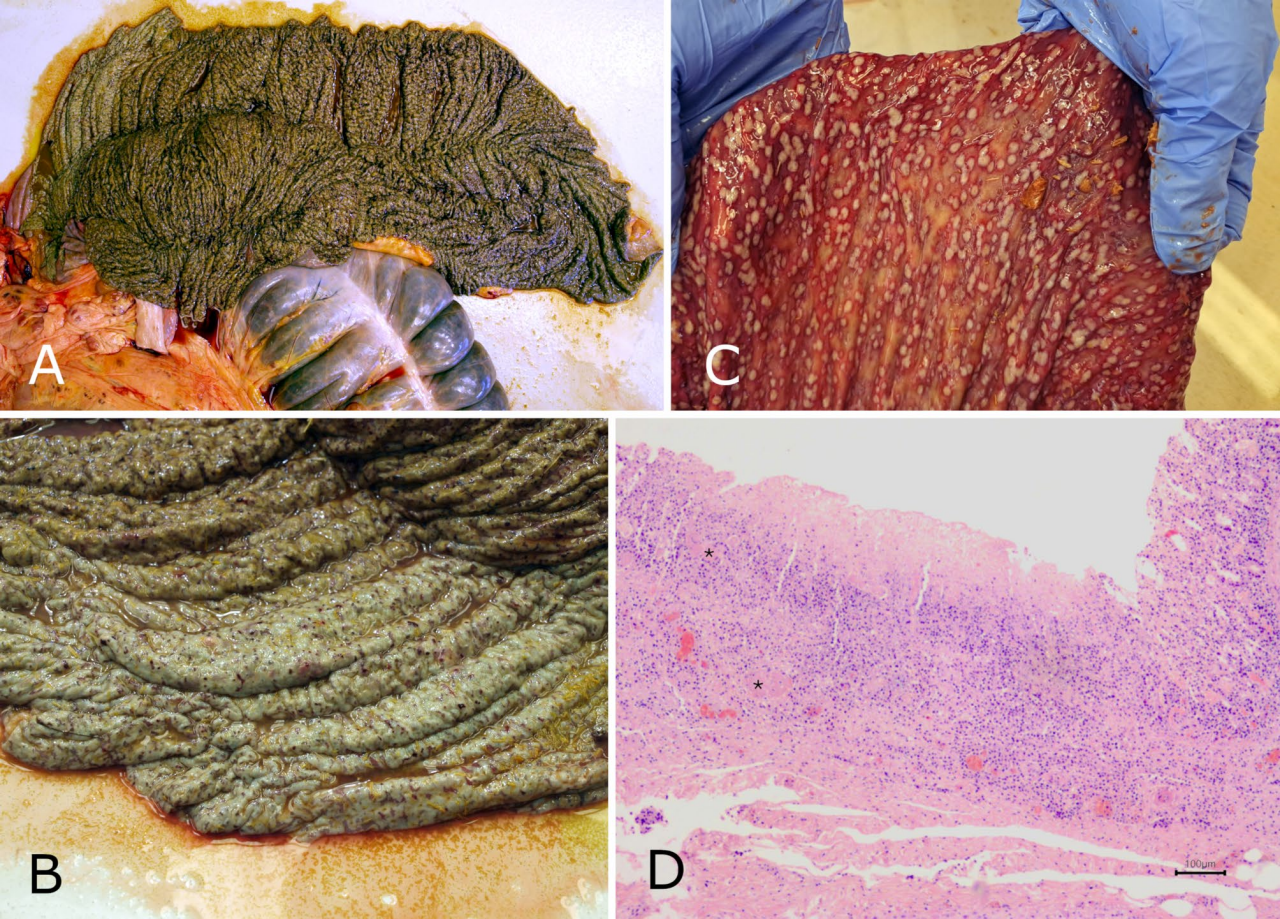
Pathological and histopathological findings (horse #1). (**A**) severe, diffuse, acute necrotic typhlitis; (**B**) closeup of part A; (**C**) severe, acute, multifocal, ulcerative colitis; (**D**) acute ulcerative colitis with marked mixed leukocytic infiltration of the submucosa and vascular thrombosis (*). Hematoxylin–eosin staining, 40X.

The pathological and histopathological examination of horses #0, #1, #2 and #5 revealed severe, acute, diffuse necrotising or necrohaemorrhagic to ulcerative entero- or typhlocolitis (Fig. 1). In the case of horse #5 multifocal necrotising hepatitis and acute necrohaemorrhagic pneumonia and multifocal chronic pleuritis was found in addition to the gastrointestinal findings. In horse #3 diffuse superficial colitis was found.

Summarized table of pathological findings and microbiological results is presented in Supplementary Material.

### Data and results of bacteriological examinations and serotyping

The faecal or *post-mortem* bacteriological samples of horses #1–5 revealed the presence of *Salmonella* spp. Four isolates were serotyped as *S.* Typhimurium (H1, H2, H4 and H5) while *S.* Coeln (H3) was identified in one case. Although *Salmonella* spp. could not be cultured from the necropsy samples, histopathological examination later supported the possibility of *Salmonella* infection in the case of horse #0. Direct PCR performed on the conserved colonic content, without prior enrichment, together with repeated bacteriological culturing, revealed the presence of *Salmonella* spp., which was serotyped as *S.* Typhimurium (H0). All *Salmonella* isolates were included in further laboratory examinations. Detailed data of necropsy and bacteriology are listed in Supplementary Material.

During the outbreak 30 environmental samples (7.5%) proved to be positive for the presence of *Salmonella* spp., and serotyping identified 17 isolates as *S.* Typhimurium, 2 isolates as *S.* Coeln and 3 as *S.* Szentes.

Two *S.* Typhimurium isolates originated from the latest confirmed (10 September 2024) positive environmental samples (E1-E2) were included in further investigations.

At the end of the intensive cleaning and disinfection period, 41 environmental samples were collected (October 9, 2024) to assess the efficacy of the measures. All of these samples proved to be negative for the presence of *Salmonella* spp.

Detailed data of the environmental samples are presented in Supplementary Material.

### Molecular epidemiology: characteristics of the *Salmonella* isolates.

#### Relatedness of the isolates

Sequencing data of *S.* Typhimurium (H0, H1, H2, H4, H5) and *S.* Coeln (H3) strains obtained in this study have been submitted to the NCBI Sequence Read Archive database under the BioProject accession number PRJNA1269797 (https://www.ncbi.nlm.nih.gov/sra/PRJNA1269797).

The *S.* Typhimurium isolates involved in the genomic analysis belonged to MLST type ST376, retrieved from the WGS data, while *S.* Coeln isolate was classified into an unrelated sequence type.

**Fig. 2:**
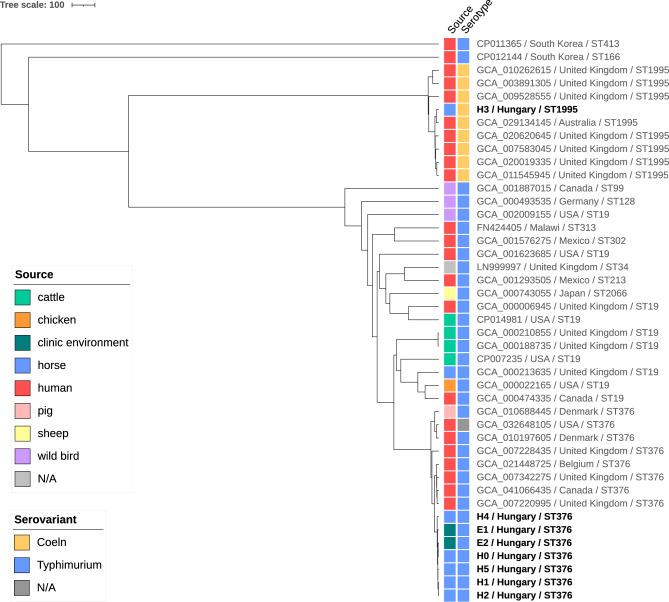
Whole genome-based similarity tree of *Salmonella* isolates. The tree was constructed using maximum core genome multilocus sequence typing results of *Salmonella* serovars isolated from the outbreak described in this study, and other *Salmonella* isolates from public databases.

When the different isolates were analysed together with reference genomes, the number of maximum core genome (mcg) MLST loci was 4,044, and our *S.* Typhimurium isolates formed a distinct, homogeneous cluster on the similarity tree (Fig. 2). Their pairwise allelic distances varied between 1 and 14, whereas the distances compared to other ST376 strains (n = 8) ranged between 31 and 78.

Although providing a higher genetic resolution, the mcgMLST analysis of only the ST376 strains did not alter this picture: across 4,256 shared loci, their allelic distances ranged from 2 to 17, while they differed from other strains by 32 to 82 alleles. Distance matrices are available in the Supplementary Material.

#### Virulence

Eleven SPIs were identified in the *Salmonella* isolates. In addition to the commonly observed SPI-1 to SPI-5, all strains carried SPI-9, and SPI-12 to SPI-14. Furthermore, marker genes of two additional pathogenicity islands, CS54 and an unnamed SPI had been detected, in the examined *S.* Typhimurium isolates.

#### Antimicrobial resistance gene analysis and antimicrobial susceptibility testing

There were marked differences in both the antimicrobial resistance gene content and phenotypic resistance profiles across the isolated *Salmonella* serovars.

*S.* Coeln isolate (H3) harboured only a single major resistance gene, an *aac(6')-Iaa* variant (pairwise identity: 97.3%). Although this gene is presumed to confer aminoglycoside resistance, the isolates were susceptible to gentamicin. *S.* Coeln exhibited phenotypic resistance to penicillin, ampicillin and tetracycline, despite lacking corresponding resistance determinants.

**Fig. 3:**
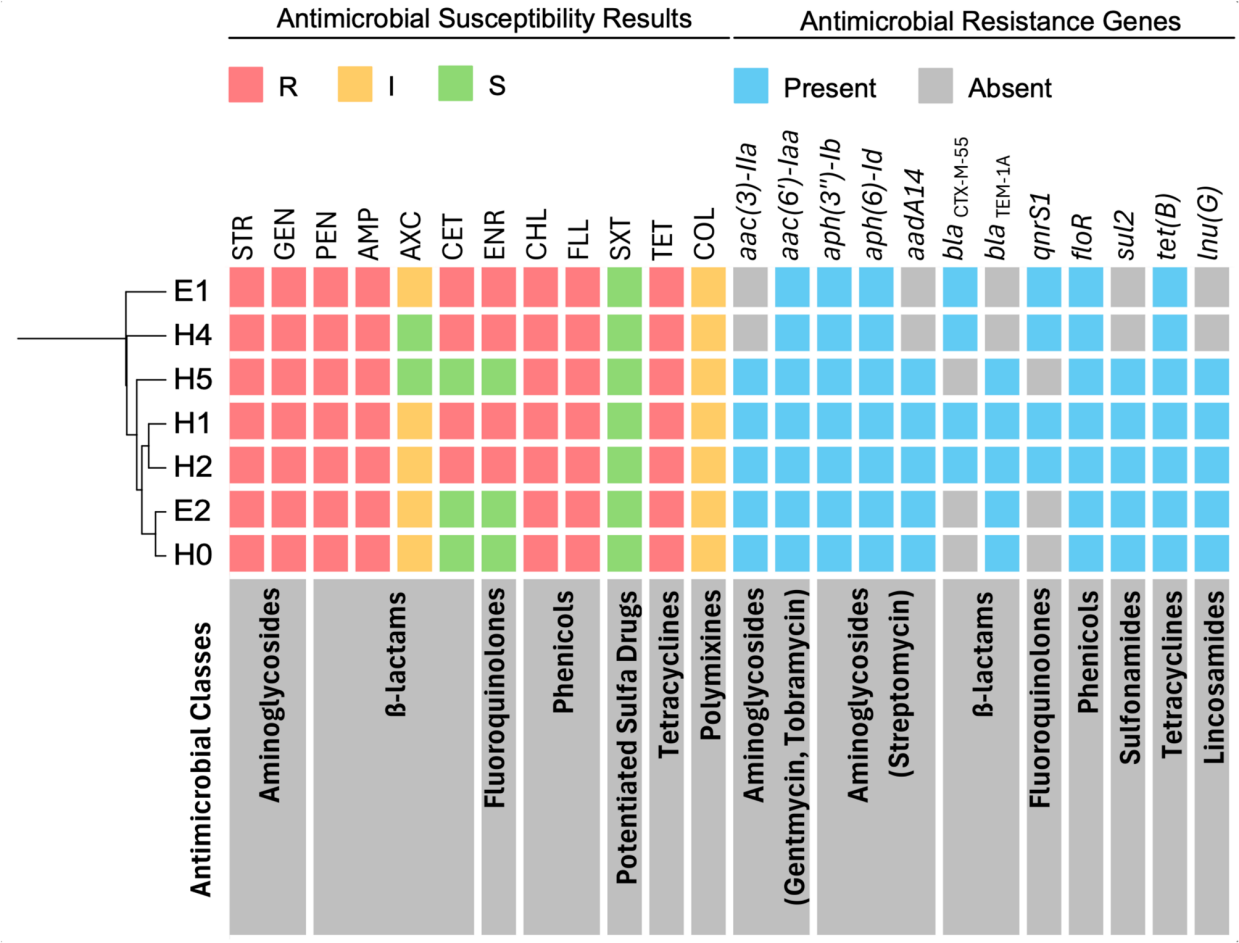
Phenotypic antimicrobial resistance and antimicrobial resistance genes of the *S.* Typhimurium isolates. H0-5 – *S.* Typhimurium isolates of horse origin; E1-2 – *S.* Typhimurium isolates of environmental origin. STR-Streptomycin, GEN-Gentamicin, PEN-Penicillin G, AMP-Ampicillin, AXC-Amoxicillin/clavulanic acid, CET-Ceftiofur, ENR-Enrofloxacin, CHL-Chloramphenicol, FLL-Florfenicol, SXT-Trimethoprim/sulfamethoxazole, TET-Tetracycline, COL-Colistin.

In contrast, *S.* Typhimurium (H0-H2, H4, H5) isolates possessed a broader array of resistance genes and exhibited more extensive phenotypic resistance, with notable variation among the isolates. Isolates H0, H5 and E2; H1 and H2; as well as H4 and E1 had identical ARG contents (Fig. 3).

Isolates H0 and E2 were multidrug-resistant, displaying the typical ACSSuT pattern (Ampicillin – Chloramphenicol – Streptomycin – Sulfamethoxazole—Tetracycline), along with gentamicin resistance (Fig. 3). Except for sulfamethoxazole, phenotypic resistance was consistent with the detected antimicrobial resistance genes. Sulfamethoxazole was tested in combination with trimethoprim, and no resistance mechanism was identified against trimethoprim, resulting in a susceptible phenotype (Supplementary Material).

All *S.* Typhimurium isolates carried at least one ESBL gene, *bla*_TEM-1A_ (H0, H5 and E2) or *bla*_CTX-M-55_ (H4 and E1) or both of them (H1 and H2). ESBL production was confirmed using the dedicated microdilution test kit only in isolates carrying the *bla*_CTX-M-55_ gene. Isolates H1, H2, H4 and E1 additionally carried *qnrS1* gene conferring resistance to fluoroquinolones (Fig. 3). Both *bla*_CTX-M-55_ and *qnrS1* genes were in close proximity to each other on a mobile genetic element belonging to the Tn3 transposon family (Fig. 3).

The isolate H4 and E1 retained the ESBL and fluoroquinolone resistance genes but lacked five other ARGs, which encode resistance to gentamicin, streptomycin, ampicillin, and sulfamethoxazole. Despite this loss, its phenotypic resistance profile remained unchanged and resembled that of other ceftiofur- and enrofloxacin-resistant isolates (H1 and H2). These missing genes were also found on a MGE in the examined *S.* Typhimurium isolates.

### Retrospective assessment of microbiological records and assessment of infection clusters

During the record-based assessment of the microbiological results (January 2010 and July 2024) of the LDC, altogether 157 horses were identified, from which at least one *Salmonella* culture was performed. The annual number of cases with *Salmonella-*selective culture varied between 3 and 18 from 2010–2023. From the 23 (14.7%) *Salmonella*-positive patients eight *Salmonella* serovars were obtained. These serovars were *S.* Kentucky (n = 9), *S.* Typhimurium (n = 5), *S.* Abony (n = 3), *S.* Bovismorbificans (n = 1), *S.* Coeln (n = 1), *S.* Enteritidis (n = 1), *S.* Kottbus (n = 1), *S.* Szentes (n = 1) and one isolate from 2013 was lost before typing (not typed, NT). Temporal distribution of the serovars is summarized in Table 1.

**Table 1:** Summary of the retrospective assessment of microbiological records of the LDC (Jan 2010 – July 2024).

Year	Annual caseload	Sampled	Positive	*S.* Kentucky	*S.* Abony	*S.* Bm.^1^	*S.* Coeln	*S.* Enteritidis	*S.* Kottbus	*S.* Szentes	*S.* Typhimurium	Not Typed
2010	657	9	0									
2011	617	6	1	1†								
2012	670	15	8	*7 (1†)							1†	
2013	661	13	1									1
2014	692	18	2		*2							
2015	510	4	1	1								
2016	638	12	0									
2017	636	8	0									
2018	825	3	0									
2019	908	15	5		1†	1†			1		*2 (1†)	
2020	997	15	1				1					
2021	1 371	16	2							1†	1	
2022	1 231	5	1								1†	
2023	1 293	13	0									
2024**	1 124	5	1					1				
*Total*	***12 830***	***157***	***23***	***9***	***3***	***1***	***1***	***1***	***1***	***1***	***5***	***1***

Sampled – number of horses, that were sampled for the presence of *Salmonella* spp. Positive – number of horses proved to be positive for the presence of *Salmonella* spp. ^1^*S.* Bovismorbificans; * case clusters; 2024**—microbiological records were reviewed until 26 July 2024; † death of an affected animal.

Three clusters of possible nosocomial infection could be defined during this period (Table 1.). One involved seven horses in 2012, all infected with *S.* Kentucky. Two further clusters involved two patients in April 2014, and two in November–December 2019, infected with *S.* Abony and *S.* Typhimurium, respectively. From the 23 *Salmonella*-positive cases, eight animals (34.8%) died or were euthanised, and the isolated serotypes were *S.* Typhimurium (n = 3), *S.* Kentucky (n = 2), *S.* Abony (n = 1*), S.* Bovismorbificans (n = 1) and *S.* Szentes (n = 1).

### Passive surveillance for the isolation of *Salmonella* spp. and assessment of infection clusters

Following the outbreak, case-based epidemiological surveillance was conducted by the LDC, between 10 October 2024 and 31 August 2025. During this period 56 at-risk-horses were sampled and 11 (19.6%) found to be positive for eight different *Salmonella* serovars. (Table 2). These were *S.* Martonos (n = 3), *S.* Szentes (n = 2), while *S.* Schleissheim, *S.* Litchfield, *S.* Enteritidis, *S.* Abony, monophasic *S.* Typhimurium and *S.* Infantis serovariants were presented with one isolate each. The three *S.* Martonos formed a well-isolated case cluster in December 2024.

**Table 2:** Summary of the results of the passive surveillance of *S.* spp. (Oct 2024 – Aug 2025).

		*S.* Martonos	*S.* Schleissheim	*S.* Litchfield	*S.* Enteritidis	*S.* Abony	m-*S.* Typhimurium	*S.* Szentes	*S.* Infantis	Negative
2024	Oct									3
	Nov									1
	Dec	*3(1†)								9
2025	Jan									3
	Feb									2
	March									1
	April		1							7
	May			1†	1†	1†				2
	June						1†	1†		9
	July									5
	Aug							1	1	3

m-*S.* Typhimurium – monophasic-*S.* Typhimurium; * case clusters; † death of an affected animal.

Six *Salmonella*-positive horses died or were euthanized. The serotypes involved in these lethal cases were *S.* Litchfield*, S.* Abony, *S.* Enteritidis, *S.* Martonos, monophasic *S.* Typhimurium and *S.* Szentes. Temporal distribution of the isolated *Salmonella *serovars is shown in Table 2.

## Discussion

Nosocomial *Salmonella* infections in equine clinics may occur either by horse-to-horse infection or via exposure to a common source (e. g.: personnel, fomites, contaminated feed). These outbreaks can result in high morbidity and mortality of the patients^6,9,13,25,45^.

In 2024, an outbreak of equine salmonellosis with a high case fatality rate occurred in a Hungarian equine clinic. We investigated the isolated *S.* Typhimurium strains by molecular genomic analysis and retrospectively evaluated the microbiological records in conjunction with the recently acquired results of passive surveillance, to place the recent outbreak into a broader epidemiological context.

At the time of admission of horse #0 (a recently imported animal, with suspected transport associated pneumonia) to the clinic, the animal was immediately isolated (23 July 2024). *S.* Typhimurium could not be detected with the applied culture technique in the intestinal samples. A plausible explanation is that the resident intestinal microbiota overgrew *Salmonella* spp. during the non-selective pre-enrichment phase^3^. However, *Salmonella* infection was included in the differential diagnostics based on the subsequent histopathological results. When *S.* Typhimurium later emerged as the causative agent of the outbreak, a repeated detection attempt was made on a frozen colonic content sample of horse #0. This time the presence of *S.* Typhimurium was successfully proved by using PCR and culture, thus identifying horse #0 as the primary case of the outbreak. In cases of possible *Salmonella* involvement the reliability of laboratory diagnostics could be increased through the parallel use of selective pre-enrichment methods and real-time PCR^35^. To improve the laboratory detection rate of *Salmonella* spp. from intestinal and reflux samples^30,35^, the use of selective pre-enrichment media were implemented promptly in routine bacteriology diagnostics of the LDC during the outbreak.

The clinical signs of fever, reflux, leukopenia and colic in horse #1 within 48 h after abdominal surgery, the rapid deterioration of the clinical condition of the patient, and the inevitable euthanasia followed by pathological findings of severe, acute diffuse necrotising typhlocolitis, suggested a possible *Salmonella* infection. During the clinical stay of horse #1, it was not admitted to isolation. The presence of *Salmonella* spp. was confirmed by bacteriology after the euthanasia of horse #1, thus this animal was identified as the index case of the outbreak (Table 1).

After the identification of the index case, the extensive environmental sampling indicated rapid spread of *S.* Typhimurium at the clinic (Supplementary Material), despite the cleaning and disinfection protocol that was implemented routinely after the discharge of each patient. Horse #0 (primary case) was immediately and routinely isolated, but horse #1 did not, the dissemination of the bacterium in the hospital environment may have resulted from multiple contamination pathways. The negative result of *Salmonella* culturing may hide the possibility of the potential presence of the agent, so the movement of the personnel and fomites could have been responsible for the contamination. The carrier rate and bacterial shedding could not be determined precisely, because only those animals were sampled that showed clinical signs suspicious of salmonellosis and a misclassification may occur when diagnostic methods with suboptimal sensitivity are applied^45^. The active surveillance for the presence of *Salmonella* spp. could facilitate the early detection of the pathogen^24^, and the gold standard bacterial culturing method should be complemented with real-time PCR examination of the samples^35^. If only patients with clinical signs suspicious of salmonellosis (e. g.: diarrhoea, fever and leukopenia) are sampled, the detection rate of *Salmonella* spp. would be 50% lower, thus allowing further spread of the pathogen in the environment^24^. The persistent environmental presence of *Salmonella* spp. could be due to the subclinical shedders, and under stress (e. g.: transportation, hospitalisation) the ratio of subclinical shedders tends to increase^19^, so an active monitoring system (e. g.: infection control program) should support the detection of *Salmonella* carrier and shedder horses.

Based on the wgMLST analysis, the *S.* Typhimurium isolates associated with the outbreak formed a distinct, seemingly homogeneous cluster on the similarity trees. Accordingly, the isolates were considered clonally related, supporting a nosocomial origin of the infections^31,32^. It should be noted, however, that the genetic distances among the outbreak isolates varied considerably (1–14 and 3–17 allelic differences). This variability, together with corresponding differences in antimicrobial resistance gene (ARG) profiles, may raise questions regarding their apparent clonality.

Several explanations may account for these observations. One potential factor is the sequencing platform used (MinION, Oxford Nanopore Technologies), which is associated with a higher base-calling error rate compared to short-read platforms and may bias allele assignment and clustering. Although recent advances in ONT chemistry and the application of carefully selected bioinformatic tools can substantially reduce inter-platform differences^46^, technical artefacts cannot be fully excluded. In addition, the use of strict filtering and polishing criteria may have resulted in the loss of certain ARG-carrying reads during downstream analyses in some genomes, contributing to the observed differences.

Furthermore, the larger number of loci included in the maximum core genome analysis (over 4,000 loci) may have contributed to higher allelic distances compared with analyses based on the predefined cgMLST scheme comprising 3,002 loci for *Salmonella enterica*^47^. Finally, in addition to technical factors, the possibility of a polyclonal outbreak cannot be fully excluded^48^, in which highly similar clones may persist in the clinical environment. As no historical *S.* Typhimurium isolates from the equine clinic outside the 2024 outbreak were whole genome analysed, this possibility would require further investigation.

Altogether eleven *Salmonella* pathogenicity islands (SPIs) were detected in the examined *S.* Typhimurium isolates, SPI-1 and SPI-2 are critical for host cell invasion and intracellular survival and are able to translocate more than 40 virulence effectors that interfere with a wide range of host cell processes^39^. SPI-2 has key role in establishing and sustaining a protective intracellular compartment (*Salmonella* containing vacuole) enhancing the survival in macrophages and through the expression of certain genes it has enhanced growth advantage over the microbiota^21,39^. SPI-3 is involved in the initial phase of host cell and bacterial interaction and also affects the long-term persistence^21^, while SPI-4 and SPI-9 directly influences and enhances the transport of certain adhesins and toxins to the extracellular space from the cytoplasm of the bacterium^39^. SPI-12 enhances the intracellular survival of the bacterium^49^, SPI-12, −13 and −14 are ubiquitous in *S. enterica* subspecies *enterica*^39,50^. The CS54 pathogenicity island contains putative outer membrane protein and was presented in the *S.* Coeln isolate as well.

Compared to previous data, showing 38–44% case fatality of *S.* Typhimurium infections^6,10^, the case fatality in the present outbreak was extremely high, four of the five affected animals succumbed or had to be euthanised. Based on the genomic analysis of the *S.* Typhimurium isolates, the observed higher virulence might be supported by virulence genes encoded by the serovar specific virulence plasmid (pSLT) and the CS54 pathogenicity island, but other factors may also play a role. Although assigning mortality to a pathogen within a highly unevenly compromised population (e. g. hospitalized equine patients) could be misleading, because several other factors might influence the outcome of a *Salmonella* infection, including the host’s immune status and the pathogen dose^45^. The exceptionally high case fatality observed in this outbreak may have been attributable not only to the virulence of the *S.* Typhimurium isolates, but also to the undefined immune status of the affected horses and the considerable environmental presence of the pathogen recorded during the middle phase of the epidemic.

MDR *Salmonella* spp., particularly ESBL-producing isolates, have been frequently identified as causative agents of severe infections in horses, posing an escalating challenge for infection control and biosafety measures in veterinary hospital environments^11,32,51^. In our ARG and AMR examinations, all *S.* Typhimurium strains proved to be MDR and all of them possessed at least one or two ESBL genes.

Isolates H4 and E1 lacked five ARGs, encoding resistance to gentamicin, streptomycin, ampicillin and sulfamethoxazole. Even with this loss, the phenotypic resistance profile did not change and was similar to that of the other ESBL-positive outbreak isolates. The loss of ARGs without corresponding change in phenotypic resistance suggests possible redundancy. It should be noted that horse #4 was the only animal that recovered from the infection. Recent ESBL outbreaks have predominantly involved the CTX-M type and more than 80 types of it have been described as widespread in hospitals, food animals, vegetables and in the environment^49^. Four *S.* Typhimurium isolates (H1, H2, H4 and E1) out of seven contained *bla*_CTX-M-55_, and exhibited phenotypic ESBL AMR pattern as well, while those isolates (H0, H5 and E2) that contained only *bla*_TEM-1A_ gene did not inactivate ceftiofur. CTX-M type ESBLs are located on extra-chromosomal mobile genetic elements and mainly disseminated through conjugative transmission^52^. Colic surgery and the perioperative application of antibiotics can lead to dysbiosis and to reduced diversity of microbiota^53^, contributing to the possible transmission of MGEs^52^, that may result in the different ARG profiles even within the same outbreak clonal lineage. The emergence of ESBL-*Salmonella* in the recent outbreak poses serious health concerns regarding personnel and also claims the implementation of a strict infection control program in the future at the equine veterinary hospital.

We retrospectively reviewed the equine *Salmonella*-related cases at the clinic back to 2010, to put the recent outbreak into a broader epidemiological perspective.

The findings of the record-based assessment and the enhanced passive survey are in accordance with previous studies that numerous *Salmonella* serotypes might emerge in equine hospitals^22,29,54^. Between January 2010 and July 2024, the presence of eight different serovars was recorded in the archives of LDC, namely *S.* Kentucky, *S.* Typhimurium, *S.* Abony, *S.* Bovismorbificans, *S.* Coeln, *S.* Enteritidis, *S.* Kottbus, *S.* Szentes, while one isolate from 2013 was not typed.

*S.* Szentes was isolated in 2021 from a horse succumbed at the clinic and *post mortem* diagnosed with an ulcerative-necrotizing colitis, while in 2020 *S.* Colen was isolated from a non-fatal equine case. The occurrence of *S.* Szentes and *S.* Coeln in the environmental samples during the recent outbreak might be the consequence of the ability of *Salmonella* spp. to survive in the environment for prolonged periods of time^7,25^. Although the emergence of *S.* Coeln in horse #3, might also be due to the latent carrier status of the pony mare at the time of hospitalisation. This assumption had not been confirmed with the comparative WGS analysis of the *S.* Szentes an *S.* Coeln strains and should be the subject of further studies.

One of the main risk factors of environmental *Salmonella* spreading is high caseload of the clinic. Compared to 2016, an increase in the annual caseload was observed in 2019 (Table 2), as well as in the number of *Salmonella-*positive cases, but no statistical analysis was carried out to investigate this correlation.

During the eleven months of the passive surveillance, *Salmonella* serovars were isolated in notably high numbers with considerable diversity. Compared to the 14.7% of *Salmonella-*positive cases observed in the retrospective assessment, the percentage of *Salmonella-*positive samples increased to 19.6% in the past year and the presence of eight different *Salmonella *serovars were identified. In six cases out of eleven, the *Salmonella-*positive animals succumbed or were euthanised.

This increase in *Salmonella-*positive cases observed through the enhanced passive surveillance could not be explained only by the application of targeted bacteriological examinations, because according to the routine bacteriological examination procedure of the LDC, *Salmonella-*testing is included in the pathological examination of carcasses exhibiting suggestive gastrointestinal lesions. However, the elevation of the numbers of *Salmonella-*positive cases could be facilitated by numerous and immeasurable latent factors, reflecting human impact – such as increased personnel demands during high caseloads and care of compromised patients – likely reduce compliance with infection control and increase opportunities for pathogen transmission within and between facilities^55^. It must be highlighted that even though infection control programmes incorporate rigorous personal and environmental hygiene practices, outbreaks can still arise, often associated with high case fatality rates and significant financial implications^55^.

Based on our recent findings, four *Salmonella-*infection clusters and one unprecedentedly highly fatal *S.* Typhimurium outbreak could be observed in the last 15 years at the equine veterinary clinic. Although *S.* Kentucky nowadays is a globally emerging clone^20^, which caused a case cluster of five horses with one fatal case (May–August 2012), this serovar had not been isolated since 2012 at the clinic. Two other case clusters of *S.* Abony (2014) and *S.* Typhimurium (2019) were revealed during the assessment of the microbiological records, with one fatal case in the 2019 cluster, further supporting the extremity of the 2024 outbreak.

Related to *S.* Typhimurium, one case cluster (2019), a highly fatal outbreak (2024) and a single case of monophasic *S.* Typhimurium infection was observed, resulted in 8 lethal cases. Which means, that *S.* Typhimurium caused the most fatality (47%) amongst the *Salmonella-*related infections at the equine clinic in the last 15 years. This corresponds well with previously published data^6,10,19^.

Our results highlight that *Salmonella* is repeatedly introduced into the equine hospital environment, and serotypes may differ markedly regarding their virulence and spreading dynamics. Appropriate biosecurity measures, including rapid and reliable diagnostics, an early warning system, and heightened vigilance might reduce the exposure of equine patients and the risk of severe outbreaks.

## Methods

### Data and clinical samples of patients associated with the outbreak

Data of equine patients that were involved in the outbreak (27 July – 27 August 2024) and that of the later identified primary case’s, were collected from the clinical records. The collected data were age, gender and breed, date of admission and release or death or euthanasia, initially reported anamnesis, clinical signs at the time of hospital enrolment, implication of abdominal surgery, admission to isolation stable and laboratory results of microbiological examinations.

### Data of environmental samples

The dates and locations of environmental swab specimen collections, submitted for laboratory detection of *Salmonella* spp. during the outbreak and the period of intensive cleaning and disinfection of the clinical environment, were recorded.

The total number of environmental samples were 440. To verify the effectiveness of infection control measures 41 environmental swab samples were collected.

All samples mentioned above were assembled by the personnel of the clinic.

### Pathology and histopathology

Necropsy of succumbed or euthanised horses was routinely carried out at the Livestock Diagnostic Centre (LDC, Department of Pathology, University of Veterinary Medicine Budapest, Üllő, Hungary). Macroscopic *post mortem* examination and sampling for histopathology and bacteriology (intestinal content and organ samples) were performed according to routine procedures of the LDC.

The tissue samples for histopathological examination were fixed in 10% neutral buffered formalin, then were routinely embedded in paraffin, cut at 4 μm and stained with haematoxylin and eosin (HE).

### Laboratory examinations

#### Bacteriological examinations, *Salmonella* PCR

All samples, including faeces, reflux and environmental swabs collected at the clinic and necropsy specimens were processed by the LDC. Specimens were pre-enriched in pepton-water (Biolab, Hungary) overnight at 37 °C and subsequently plated onto Modified Semisolid Rappaport Vassiliadis agar (Biolab, Hungary) and incubated aerobically at 41 °C for 24 h. In case of visible motility, the isolates were subcultured on *Salmonella-Shigella* agar (Biolab, Hungary) at 37 °C for a further 24 h. Necropsy samples were parallelly cultured on Columbia 5% sheep blood agar and on MacConkey agar (Biolab, Budapest, Hungary) at 37 °C for 24 h, under both normal atmospheric and anaerobic conditions.

Isolates were identified on genus level based on their colony morphology, basic biochemical tests and Gram-staining. The species and subspecies level identification was carried out using API 20E biochemical test kit (bioMérieux, Belgium). The *Salmonella* culture negative equine faeces and necropsy samples were investigated further by applying a real-time PCR test method directly on the samples, without a pre-enrichment step^56^.

#### Serotyping of the *Salmonella* isolates

Serotyping of the isolated *Salmonella* strains was carried out at the National Microbiological Reference Laboratory of the National Food Chain Safety Office (Budapest, Hungary) according to the Kauffmann-White scheme.

#### Whole genome sequencing (WGS) and bioinformatic analysis

*S.* Typhimurium strains of equine (n = 5) and environmental (n = 2) origin, as well as an equine-derived *S.* Coeln (n = 1) isolate were whole genome sequenced. The whole genome sequencing and the bioinformatic analysis were carried out at the Department of Microbiology and Infectious Diseases, University of Veterinary Medicine Budapest (Budapest, Hungary).

Bacterial DNA was extracted with the Quick-DNA Fungal/Bacterial Miniprep Kit (Zymo Research) according to the manufacturer’s instructions. The DNA quantity was measured using a Qubit 4.0 Fluorometer (Invitrogen, Life Technologies, Carlsbad, CA, USA).

We prepared the sequencing library with 400 ng of DNA following the recommendations of the Ligation sequencing gDNA by Native Barcoding Kit 24 V14 (SQK-NBD114.24, Oxford Nanopore Technologies, Oxford, UK), than loaded the prepared library onto an R10.4.1. MinION flow cell (Oxford Nanopore Technologies). We generated raw data for 72 h using a MinION mk1b instrument. Basecalling (with super accuracy basecalling mode) and demultiplexing of the long-read raw sequences were performed with Dorado v7.6.8 as implemented in MinKNOW v 24.11.10.

As the first step of long read preprocessing, we excluded the reads of the DNA control strand using NanoLyse v1.2.1^57^. We used NanoFilt v2.8.0 to trim 50 bp of reads at both the 5′ and 3′ ends to ensure that all adaptor sequences were removed and to exclude reads with a mean quality of less than 8 or with a length of less than 500 bp^57^. Finally, we evaluated and visualized the read quality metrics using NanoPlot v1.43.0^57^.

We performed de novo assembly using Flye v2.9.5-b1801^58^. All assemblies were successively polished using minimap2 v2.28-r1209, racon v1.5.0, medaka v2.0.1 (https://github.com/nanoporetech/medaka) using the r1041_e82_400bps_sup_v4.2.0 model^59,60^. We checked the contiguity and completeness of the genomes with QUAST v5.0.2 and BUSCO 5.8.2 using the BUSCO gene set of –auto-lineage^61,62^. Classical multilocus sequence types (ST) were retrieved from MLST (https://github.com/tseemann/mlst). The following tools were used for in silico speciation (KmerFinder 3.2)^63^, for sero- and genotyping (SeqSero2 1.1.0, SalmcgMLST v1.0)^64,65^.

The whole genome (wg)MLST analysis was performed using the module *Build_PGAdb* on the software PGAdb-builder^66^ for creating a PGAdb allelic profile from two sets of the genomes. The first analysis comprised all genomes, the second only the *S.* Typhimurium ST376 isolates. In this latter analysis an ST19 genome (GCA_000474335) was used as an outlier. The wgMLST trees were constructed using the *Build_wgMLSTtree* module by PGAdb database. We used the genome contigs and reference genomes as input files. Reference genomes were retrieved from Enterobase database, in the “.fasta” format^67^. Alignment coverage and identity ≥ 95% were used as parameters for PGAdb^66^. The similarity trees were visualized and annotated with sample metadata and specific genetic characteristics using the Interactive Tree of Life (iTOL) web tool^68^. We identified antimicrobial resistance genes (ARGs) and virulence factors in the complete genome sequences using ABRicate based on the ResFinder, and VFDB databases [https://github.com/tseemann/abricate]. Contigs bearing at least one ARG were screened for integrative mobile genetic elements (iMGE) using MobileElementFinder 1.0.5^69^. Then, we checked the distance between the coordinates of ARGs and iMGEs located on the same contig and defined them as linked if their distance was less than 1000; 10,000 and 50,000 base pairs (bps). To evaluate the chance of spreading ARGs, the plasmid origin of contigs with ARGs linked to iMGEs was predicted by using PlasFlow 1.1^70^. *Salmonella* Pathogenicity Islands were detected by the SPIFinder, the web services of the Center for Genomic Epidemiology for *Salmonella* available at (https://cge.cbs.dtu.dk/services/SPIFinder/) with threshold of 95% and minimum length of 60%^71^.

#### Antimicrobial susceptibility testing

To confirm the antimicrobial resistance gene profile, the phenotypic antimicrobial susceptibility of all equine-derived *S.* Typhimurium strains (n = 5), along with two isolates cultured from environmental samples were determined by using a commercially available microdilution test plate (Micronaut-S E1-318–100; Bruker, Germany). The results of the microdilution panel were augmented by assessing the isolates’ susceptibility to chloramphenicol and streptomycin, using the disc diffusion method, in accordance with CLSI (Clinical and Laboratory Standards Institute) standards^72^. The presence and activity of the ESBL (*bla*_CTX-M-55_) detected in certain *S.* Typhimurium isolates was also investigated on representative carrier and non-carrier isolates by using the “Micronaut-S ß-lactamases” microdilution plate (E111-040; Bruker, Germany). The susceptibility test results were evaluated either automatically by the Bruker’s software in case of the microdilution test kits or else according to CLSI^72^. The complete list of antimicrobials tested, MIC (Minimum Inhibitory Concentration) values, and zone diameters are available in the (Supplementary Material).

### Retrospective assessment of microbiological records and assessment of infection clusters

The microbiological results recorded between January 2010 and July 2024 by the LDC were reviewed. All equine clinic-related horse cases were selected where *Salmonella-*selective culture was performed, either from live animal samples or from necropsy specimens. Bacterial examinations were initiated by the attending veterinarian of the hospital based on the clinical signs or else by the pathologist veterinarian observing lesions suggestive of salmonellosis. The *Salmonella-*selective culture and serotyping techniques were the same as described above. Multiplicated results from the same animals were identified and consolidated into a single record. If serotyping was not performed from a necropsy sample, the isolated *Salmonella* spp. was considered the same serotype as determined in the clinical sample of the same animal.

Case clusters were determined if i) the same *Salmonella* serovariant was identified ii) in at least two horses iii) for which hospitalization overlapped in time^73^. Besides serotyping, no genomic analysis was performed to investigate the relatedness of the previously isolated *Salmonella* spp.

*Passive surveillance for the isolation of* Salmonella *spp. and assessment of infection clusters.*

Following the outbreak, enhanced passive surveillance was initiated by the LDC for the presence of *Salmonella* spp. in equine patients (10 October 2024–31 August 2025). Samples from animals or carcasses suspicious to *Salmonella* infection (e.g., clinical symptoms of colic or other gastrointestinal disease, or any kind of macroscopic lesions in the gastrointestinal tract) underwent selective-enrichment culturing, and serotyping was performed in the case of all *Salmonella* spp. isolates. Positive cases were reported immediately to the hospital.

Case clusters were determined as described above.

## Supplementary Information

Supplementary Information.

## Data Availability

The datasets supporting the conclusions of this article are included within the article and its online additional files, and also available under the PRJNA1269797 (https://www.ncbi.nlm.nih.gov/sra/PRJNA1269797) BioProject accession number of the NCBI Sequence Read Archive database. Further data are available from the corresponding author on reasonable request.
